# Supplementary material for: “FastCheckFLI PPR-like”—A Molecular Tool for the Fast Genome Detection of PPRV and Differential Diagnostic Pathogens
Source: Viruses. 2020 Oct 29;12(11):1227. doi: 10.3390/v12111227 (PMC7694148; doi:10.3390/v12111227)
Supplement: Supplementary file 1 [file viruses-12-01227-s001.pdf]

Supplementary material

Table S1. Extraction time of various protocols on the BioSprint 15 platform for original and short protocols.

Step	VET and CADOR kit			CORE kit		
	**Original**	**Short 4**	**Short 5**	**Original**	**Short 4**	**Short 5**
Lysis and binding	4 min 50 s	1 min 48 sec	1 min 48 s	14 min 12 s	1 min 48 sec	1 min 48 s
Collect beads	–	-	–	58 s	-	–
1st washing step	2 min 12 s	1 min 05 s	57 s	2 min 38 s	1 min 04 s	57 s
2nd washing step	2 min 12 s	1 min 05 s	57 s	1 min 41 s	1 min 04 s	57 s
3rd washing step	2 min 12 s	1 min 05 s	57 s	–	-	–
Drying of the beads	4 min 00 s	1 min 00 s	1 min 00 s	4 min 00 s	1 min 00 s	1 min 00 s
Elution	1 min 56 s	44 s	42 s	11 min 16 s	1 min 04 s	1 min 04 s
Release beads	26 s	26 s	26 s	17s	26 s	26 s
**Total duration**	**17 min 48 s**	**7 min 13 s**	**6 min 47 s**	**35 min 2 s**	**6 min 26 s**	**6 min 12 s**

Table S2. Reagents (in µl) and filling scheme for the rapid nucleic acid extraction protocol of three extraction kits.

	**VET kit**	**CADOR kit**	**CORE kit**
**Sample lysis**			
sample	100	100	100
lysis solution	100	100	350
**Binding**			
binding solution	350	400	350
beads	20	25	20
internal control	10	10	10
**Wash solution 1**	600	700	500
**Wash solution 2**	600	700	500
**Wash solution 3**	600	750	-[1]
**Elution buffer**	100	100	100

[1]washing step 3 is not included according to the manufacturer-specific recommendations.

Table S3. Test series based on a rapid extraction protocol: results of a comparative validation of different extraction kits regarding their runability in original protocol ("original") and maximal speed-optimized protocols ("short 4" and "short 5").

	**VET kit***			**CADOR kit***			**CORE kit***		
	**Original**	**Short 4**	**Short 5**	**Original**	**Short 4**	**Short 5**	**Original**	**Short 4**	**Short 5**
10^{-1}	21.8	22.3	23.5	22.1	22.2	25.8	20.4	23.0	23.3
10^{-2}	26.2	26.8	28.8	26.0	26.1	30.2	25.6	26.2	26.6
10^{-3}	29.5	30.2	31.7	29.1	30.0	30.1	29.0	30.6	31.2
10^{-4}	31.4	33.8	32.5	32.0	34.0	35.0	32.0	34.1	34.1
10^{-5}	36.0	37.2	35.6	35.9	37.0	38.0	35.6	No Cq	38.7
10^{-6}	38.1	38.3	No Cq	No Cq	No Cq	38.1	No Cq	No Cq	No Cq
10^{-7}	-	No Cq	No Cq	No Cq	No Cq	No Cq	No Cq	No Cq	No Cq
**Time**	**17 min 48 s**	**7 min 13 s**	**6 min 47 s**	**17 min 48 s**	**7 min 13 s**	**6 min 47 s**	**35 min 2 s**	**6 min 26 s**	**6 min 12 s**

*Extraction kits used are: VET kit = NucleoMag® VET (Macherey-Nagel, Düren, Germany); CADOR kit = MagAttract® 96 cador Pathogen Kit (Indical, Leipzig, Germany); CORE kit = MagMAX™ CORE Nucleic Acid Purification Kit (ThermoFisher Scientific, Waltham, USA); - = not tested.

Table S4. Results (Cq-values) of the device test with five qPCR cyclers using two PPRV-specific assays (Polci-mix, PPRV-mix 6): standard protocol compared to the short protocol 5.

	**Standard protocol**					**Short protocol 5**				
	**CFX96 Touch**	**Aria Mx**	**MIC**	**Light Cycler 2.0**	**Light Cycler 96**	**CFX96 Touch**	**Aria Mx**	**MIC**	**Light Cycler 2.0**	**Light Cycler 96**
**Polci-mix**										
10^{-1}	22.1	20.2	22.3	23.8	18.3	27.0	24.8	26.5	> 35.0	24.2
10^{-2}	25.6	23.3	25.4	26.9	22.4	30.7	28.6	30.3	> 35.0	27.8
10^{-3}	29.3	26.0	28.7	31.2	26.7	34.2	32.2	33.8	No Cq	31.1
10^{-4}	32.5	29.3	32.4	33.7	30.8	38.2	35.9	37.3	No Cq	34.3
10^{-5}	36.9	33.2	37.1	34.6	35.9	No Cq	38.6	No Cq	No Cq	37.7
10^{-6}	No Cq	No Cq	No Cq	No Cq	35.2	No Cq	No Cq	No Cq	No Cq	No Cq
10^{-7}	No Cq	No Cq	No Cq	No Cq	No Cq	No Cq	No Cq	No Cq	No Cq	No Cq
**PPRV-mix 6**										
10^{-1}	23.0	19.8	20.9	23.6	20.8	26.8	24,3	24.3	29.1	24.3
10^{-2}	26.3	22.5	24.3	25.6	24.3	30.0	27,8	27.7	34.3	27.4
10^{-3}	29.7	26.2	27.9	28.3	27.7	33.7	30,6	31.3	> 35.0	30.8
10^{-4}	33.0	26.7	31.2	31.0	31.8	37.4	34,7	34.6	> 35.0	33.9
10^{-5}	37.1	32.0	34.2	32.6	34.6	No Cq	38,5	No Cq	No Cq	No Cq
10^{-6}	No Cq	No Cq	37.7	33.5	37.7	No Cq	No Cq	No Cq	No Cq	No Cq
10^{-7}	No Cq	No Cq	No Cq	No Cq	No Cq	No Cq	No Cq	No Cq	No Cq	No Cq
**Time**	**1 h 38 min**	**1 h 32 min**	**1 h 38 min**	**1 h 23 min**	**1 h 34 min**	**38 min**	**33 min**	**39 min**	**25 min**	**34 min**

Table S5. Test series for a high-speed RT-qPCR: development of the Cq-values on the BioRad CFX96 with regard to a time-optimized RT-qPCR protocol ("short") compared to a standard protocol ("standard") when using two primer-probe mixtures.

	**Assay of Polci et al., 2015**			**PPRV-mix 6**		
	**Standard**	**Short 4**	**Short 5**	**Standard**	**Short 4**	**Short 5**
10^{-1}	17.3	20.7	22.4	18.7	20.7	21.4
10^{-2}	21.0	24.1	26.2	22.3	24.4	25.5
10^{-3}	24.6	27.9	30.1	25.7	27.9	28.9
10^{-4}	28.0	31.1	34.1	29.0	31.2	32.0
10^{-5}	32.1	35.3	37.7	33.4	35.4	No Cq
10^{-6}	36.6	38.8	No Cq	36.8	No Cq	No Cq
10^{-7}	No Cq	No Cq	No Cq	No Cq	No Cq	No Cq
**Time**	**1 h 38 min**	**39 min**	**35 min 2 s**	**1 h 38 min**	**39 min**	**35 min 2 s**

Table S6. Test series for a high-speed RT-qPCR: development of the RFU values on the BioRad CFX96 with regard to a time-optimized RT-qPCR protocol ("short") compared to a standard protocol ("standard") when using two primer-probe mixtures.

	**Assay of Polci et al., 2015**			**PPRV-mix 6**		
	**Standard**	**Short 4**	**Short 5**	**Standard**	**Short 4**	**Short 5**
10^{-1}	9906	9729	9131	9193	8457	7114
10^{-2}	10461	9558	7330	9266	7596	5466
10^{-3}	9976	9187	7376	10023	6600	4750
10^{-4}	10207	8034	4507	9034	4324	2499
10^{-5}	9984	4391	1678	6109	1475	492
10^{-6}	8788	1515	-1.6	4777	202	91
10^{-7}	-3.8	-9,8	-2.4	5.52	16	13
**Time**	**1 h 38 min**	**39 min**	**35 min 2 s**	**1 h 38 min**	**39 Min**	**35 min 2 s**

Table S7. Results (Cq-values) of the test series with three various lyophilized test kits: comparison of an optimal PCR-protocol ("optimal") for the validation of the dilution series, the standard protocol ("standard protocol") of the lyophilized kit and the speed-optimized protocol ("short protocol 5") of the corresponding kit; AgPath = AgPath-ID™ One-Step RT-PCR Reagents; Qiagen = QuantiTect Multiplex-PCR Kit NO ROX; lyophilised kits: Takyon™ Dry No Rox One-Step RT Probe MasterMix kit; Qscript lyo 1-step; CAPITAL™ qRT-PCR Probe Mix.

	Optimal	Standard protocol			Short protocol 5		
**PPRV**	**AgPath kit**	**Takyon kit**	**Qscript kit**	**Capital kit**	**Takyon kit**	**Qscript kit**	**Capital kit**
10^{-1}	19.3	21.4	19.1	19.9	27.1	21.3	21.1
10^{-2}	22.8	25.3	22.3	23.2	31.0	24.7	24.6
10^{-3}	26.5	28.3	25.7	26.5	34.3	28.2	28.2
10^{-4}	30.0	33.3	29.0	30.3	39.0	32.0	31.7
10^{-5}	33.4	37.5	32.7	33.3	No Cq	35.0	35.2
10^{-6}	34.9	No Cq	35.2	37.6	No Cq	37.9	No Cq
10^{-7}	No Cq	No Cq	No Cq	No Cq	No Cq	No Cq	No Cq
**Mccp**	**AgPath kit**	**Takyon kit**	**Qscript kit**	**Capital kit**	**Takyon kit**	**Qscript kit**	**Capital kit**
10^{-1}	21.2	21.4	21.8	21.8	32.6	22.1	21.9
10^{-2}	24.8	24.7	25.1	25.0	33.5	25.2	24.8
10^{-3}	28.3	28.1	28.3	28.3	37.4	28.5	28.2
10^{-4}	31.9	31.3	31.6	31.8	No Cq	31.9	31.8
10^{-5}	35.5	34.3	34.7	35.4	No Cq	35.7	34.5
10^{-6}	No Cq	No Cq	38.2	39.1	No Cq	No Cq	36.8
10^{-7}	No Cq	No Cq	No Cq	No Cq	No Cq	No Cq	No Cq
**FMDV**	**AgPath kit**	**Takyon kit**	**Qscript kit**	**Capital kit**	**Takyon kit**	**Qscript kit**	**Capital kit**
10^{-1}	22.0	27.6	22.1	21.8	31.6	23.8	22.7
10^{-2}	25.2	30.2	25.6	25.0	34.4	27.0	26.4
10^{-3}	28.5	34.1	28.9	28.1	38.7	30.0	29.6
10^{-4}	31.7	38.8	32.2	31.4	No Cq	33.3	33.0
10^{-5}	34.6	No Cq	34.5	33.5	No Cq	36.7	35.1
10^{-6}	37.9	No Cq	36.5	38.7	No Cq	37.0	No Cq
10^{-7}	No Cq	No Cq	No Cq	No Cq	No Cq	No Cq	No Cq
**GTPV**	**Qiagen kit**	**Takyon kit**	**Qscript kit**	**Capital kit**	**Takyon kit**	**Qscript kit**	**Capital kit**
10^{-1}	20.6	19.5	20.3	20.0	22.1	20.3	20.0
10^{-2}	23.7	23.2	23.6	23.2	25.6	23.9	23.2
10^{-3}	27.1	26.3	26.8	26.4	28.7	27.1	26.5
10^{-4}	30.4	29.9	30.1	30.0	32.3	30.6	30.0
10^{-5}	33.5	33.7	32.7	33.8	36.3	34.0	33.2
10^{-6}	37.3	37.1	37.0	36.2	39.2	36.3	36.5
10^{-7}	No Cq	No Cq	No Cq	No Cq	No Cq	No Cq	No Cq
**Orf**	**Qiagen kit**	**Takyon kit**	**Qscript kit**	**Capital kit**	**Takyon kit**	**Qscript kit**	**Capital kit**
10^{-1}	20.6	21.4	22.1	23.0	26.0	21.9	23.1
10^{-2}	23.9	24.7	25.3	26.2	29.2	25.2	26.2
10^{-3}	27.2	28.1	28.5	29.6	32.6	28.6	29.6
10^{-4}	30.8	31.3	32.0	32.7	36.2	32.4	33.1
10^{-5}	33.5	34.3	36.2	34.3	38.4	35.7	35.8
10^{-6}	36.2	No Cq	37.7	No Cq	No Cq	37.6	No Cq
10^{-7}	No Cq	No Cq	No Cq	No Cq	No Cq	No Cq	No Cq
